# 

**Published:** 1892-11-12

**Authors:** 


					II TRTCTE I WUKtlHUt.
7'320, Vol. XIII.?Nov. 12th, 1892.
,R?8utered at the General Post Office as a Nownpacer for
Per Annum, 8s. 6d., post free.
Monthly Farts, 6d.; post free, 8d. 1
utiiturai l UBb uutuo ni n Newspaper for Mb* t 1 ' 3 _ Ditto per Annum, 8s-, post free.
"?Umiaaion in the United Kingdom and Abroad. |
XIII.] Saturday,
November 12th, 1892.
TTwopknor.

				

